# Cost-Effectiveness Analysis of Recombinant Zoster Vaccine at Age 50 for Chinese Adults with Mild Cognitive Impairment: A Modelling Study

**DOI:** 10.3390/vaccines14050406

**Published:** 2026-05-01

**Authors:** Yifei Wu, Yao Yao, Jue Liu

**Affiliations:** 1 Department of Epidemiology and Biostatistics, School of Public Health, Peking University, No. 38, Xueyuan Road, Haidian District, Beijing 100191, China; 2 School of Public Health, Peking University, No. 38, Xueyuan Road, Haidian District, Beijing 100191, China; 3 Center for Healthy Aging Transdisciplinary Sciences, China Center for Health Development Studies, Peking University, No. 38, Xueyuan Road, Haidian District, Beijing 100191, China; 4 Key Laboratory of Epidemiology of Major Diseases (Peking University), Ministry of Education, No. 38, Xueyuan Road, Haidian District, Beijing 100191, China; 5 Institute for Global Health and Development, Peking University, No. 5 Yiheyuan Road, Haidian District, Beijing 100871, China

**Keywords:** herpes zoster, recombinant subunit vaccine, mild cognitive impairment, dementia, cost-effectiveness analysis

## Abstract

**Background**: The recombinant zoster vaccine (RZV) has been shown to reduce the risk of dementia and delay cognitive decline. However, economic evaluations in populations with mild cognitive impairment (MCI), particularly those incorporating cognitive outcomes, remain unavailable. This study evaluated the cost-effectiveness of RZV vaccination at age 50 among Chinese adults with MCI. **Methods**: A decision tree–Markov model was developed from a societal perspective to assess the lifetime cost-effectiveness of RZV (Shingrix, GSK) in a cohort of 1 million immunocompetent Chinese adults with MCI receiving vaccination at the age of 50. The primary outcome was the incremental cost-effectiveness ratio (ICER), while secondary outcomes included cases averted and the number needed to vaccinate (NNV) to prevent one case of herpes zoster (HZ), postherpetic neuralgia (PHN), and dementia. A willingness-to-pay (WTP) threshold equivalent to the 2024 Chinese gross domestic product (GDP) per capita (13,121 USD) was applied. Sensitivity analyses were conducted to test the robustness of the results. **Results**: Over a lifetime horizon, RZV vaccination was estimated to avert 54.64% of HZ cases, 97.58% of PHN cases, and 12.28% of dementia cases compared with no vaccination, resulting in an additional 2.23 million quality-adjusted life years (QALYs) gained. The ICER was 4216.99 USD/QALY, remaining well below the WTP threshold. The corresponding NNVs were 6.25 for HZ, 24.03 for PHN, and 280.82 for dementia progression. **Conclusions**: RZV vaccination is cost-effective for Chinese adults aged 50 years with MCI, providing substantial health gains through reductions in both HZ burden and dementia progression.

## 1. Introduction

Herpes zoster (HZ) results from the reactivation of the varicella-zoster virus (VZV) latent within the sensory ganglia [1]. The age-related decline in VZV-specific cellular immunity, termed immunosenescence, increases the incidence and recurrence of HZ and its complications [2,3,4]. Postherpetic neuralgia (PHN) remains the most frequent chronic complication; its persistence for months or years substantially impairs quality of life and imposes considerable economic burdens on households and healthcare systems [1,5,6].

Epidemiological evidence links VZV infection to cognitive impairment in older adults. Emerging evidence suggests that VZV reactivation may trigger neuroinflammatory processes that accelerate neurodegeneration and increase the risk of progression to dementia [7,8]. Mild cognitive impairment (MCI) represents the transition from normal aging to dementia and serves as an interventional window to delay progression [5,9,10]. Individuals with MCI may be particularly vulnerable to the adverse consequences of HZ, as HZ episodes in this population cause cutaneous and neurological injury while aggravating cognitive decline through widespread inflammation, resulting in a compounded and multifaceted disease burden [4,8,11,12].

The recombinant zoster vaccine (RZV) prevents HZ and its complications. Global trials, including the ZOE-50 and ZOE-70 studies, reported protective efficacies of 97.2% and 91.3% in individuals aged ≥50 and ≥70 years, respectively [13,14,15]. China approved RZV (London, United Kingdom, Shingrix, GSK) in 2020 for immunocompetent adults aged ≥50 years [16]. Beyond preventing HZ, quasi-experimental and cohort studies evidence the effect of RZV on cognitive decline. A natural experiment indicated that RZV increased dementia-free survival by 17% compared to the live-attenuated vaccine (ZVL) [17]. A retrospective study of individuals aged 65 years and older in the United States reported that vaccination with two doses of RZV was associated with a 51% reduction in the risk of dementia [18]. These data suggest RZV may confer dual benefits by both preventing HZ and modifying neurodegenerative trajectories.

Economic assessments of RZV in the general population have been well established [19,20,21,22,23]. However, evaluations specifically for the MCI cohort that incorporate cognitive outcomes remain unavailable. This population deserves separate analysis because individuals with MCI face an elevated susceptibility to HZ and a high baseline risk of progressing to dementia [8,9]. Existing economic evaluations typically assume homogeneous baseline risks and do not account for the elevated susceptibility to HZ, accelerated cognitive decline, and higher downstream care costs associated with MCI, and are therefore not directly generalizable to this high-risk subgroup [8,19,20,21,22,23]. Moreover, prior models have largely focused on HZ-related outcomes and have not incorporated potential cognitive benefits, which may lead to an underestimation of the full value of vaccination in populations at risk of dementia progression [4,11,12,24].

Given the substantial clinical and economic consequences of dementia progression and the potential for vaccination to influence both infectious and neurodegenerative pathways, a dedicated economic evaluation in individuals with MCI is warranted. By jointly considering HZ, PHN, and dementia-related outcomes, such an analysis can better capture the full value of vaccination and provide more relevant evidence for decision making. This study, therefore, developed a decision tree–Markov model to assess the long-term cost-effectiveness of RZV at age 50 for Chinese adults with MCI from a societal perspective, to inform vaccination policy and resource allocation in the context of population aging.

## 2. Materials and Methods

### 2.1. Study Design

This analysis employed a decision tree–Markov model from a societal perspective to assess the cost-effectiveness of the RZV among adults aged 50 years with MCI. The model simulated an initial cohort of 1 million immunocompetent Chinese adults aged 50 years, all of whom had MCI at baseline. The model was not stratified by sex, and all parameters were applied as population-average estimates across males and females. Age 50 was selected as the baseline because it represents the minimum age for which RZV is approved in China and the earliest point at which vaccination can be initiated [16]. This modelling approach reflected a cohort initiating vaccination at the point of eligibility and allowed estimation of lifetime health and economic outcomes from the time of vaccination. Utilizing a lifetime horizon starting from this earliest age of eligibility ensured the comprehensive capture of long-term clinical benefits, cumulative costs, and quality-adjusted life year (QALY) gains associated with vaccination. A 1-year cycle length was utilized over a lifetime horizon extending to age 100 to capture the long-term influence of RZV on HZ, cognitive outcomes, and survival. Although the model was initialized at a single age, the results can be interpreted as representing the long-term cost-effectiveness of vaccination when administered at the earliest eligible age. The reporting of this study adhered to the Consolidated Health Economic Evaluation Reporting Standards (CHEERS) statement [25].

### 2.2. Model Structure

**Figure 1:**
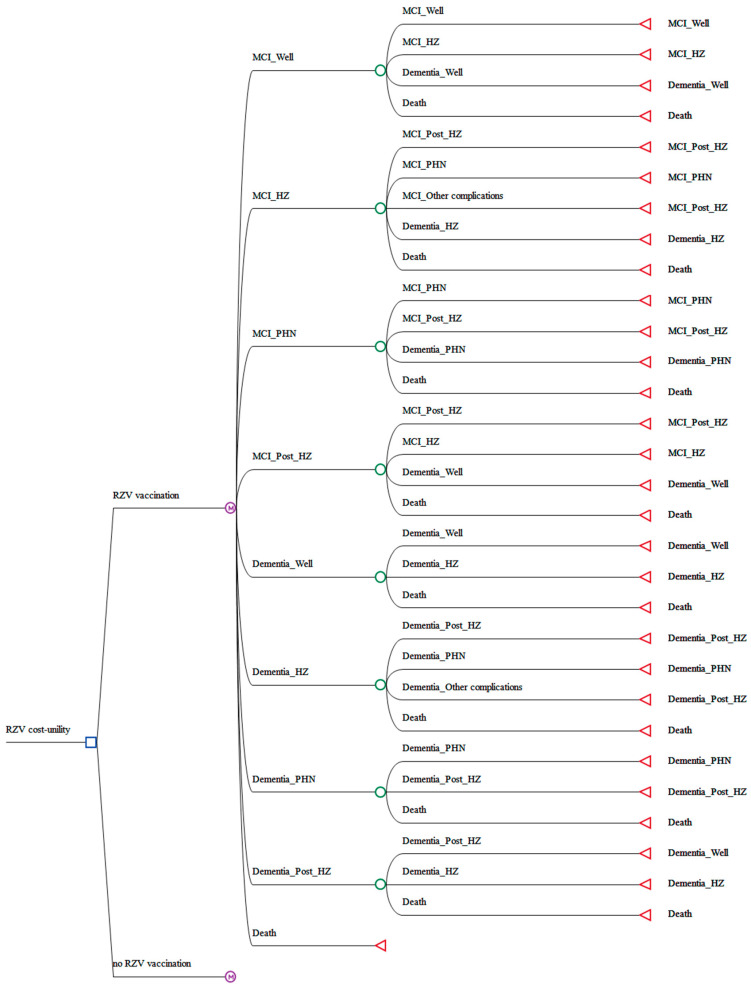
Overview of the decision tree–Markov model. HZ, herpes zoster; PHN, postherpetic neuralgia; MCI, mild cognitive impairment; RZV, recombinant zoster vaccine.

The study compared two strategies, no vaccination and RZV vaccination, in which individuals received a complete two-dose series. No vaccination was selected as the primary comparator, as it reflects the current status quo among older adults in China, where shingles vaccination is self-paid, and coverage remains extremely low [26]. Evaluating RZV against this baseline is therefore essential for assessing the socio-economic feasibility of introducing an organized vaccination program. A decision tree–Markov model was constructed using TreeAge Pro 2022 (TreeAge Software Inc., Williamstown, MA, USA), as shown in Figure 1. The potential reversion from MCI to normal cognition was not modelled in accordance with the principle of modelling parsimony. The model comprised nine mutually exclusive and collectively exhaustive health states defined by VZV infection status and cognitive progression. Within the MCI stage, four health states were specified: MCI without HZ (MCI_Well), acute HZ (MCI_HZ), PHN (MCI_PHN), and recovery state following HZ (MCI_Post_HZ). Corresponding health states were defined for the dementia stage, and one absorbing state for death. Transitions were allowed between HZ-related health states, as well as from MCI to dementia. Complications other than PHN, including ophthalmic, auditory, and neurological complications, were modelled as acute events (MCI_Other complications) rather than as distinct health states. The model employed a 1-year cycle length and a lifetime horizon, defined as up to 100 years of age or death. A half-cycle correction was applied.

### 2.3. Epidemiological Data

All-cause mortality was obtained from the China Statistical Yearbook 2025 and the China Population Census Yearbook 2020 [27,28]. The bidirectional annual transition probabilities between MCI and normal cognition were derived from a longitudinal study with 15 years of follow-up that utilized a multi-state Markov transition analysis [29]. And the annual transition probability from MCI to dementia was derived from expert consensus and cohort studies [5]. Additionally, the annual transition probability of MCI progressing to death was estimated using data from a Chinese prospective cohort study [30], whereas the annual transition probability of dementia progressing to death was obtained from a meta-analysis that included 261 studies and covered more than 5.55 million participants [31]. Age-specific incidence and recurrence rates of HZ were derived from a meta-analysis of Chinese cohort studies [32] and electronic health record data [33]. Due to the lack of relevant data in China, the case-fatality rate of HZ was informed by an economic evaluation conducted in a Japanese population, assuming a degree of clinical comparability in HZ outcomes among elderly populations within the East Asian region [21,22]. Estimates of PHN incidence, recovery rates, and probabilities of other complications were obtained from epidemiological studies [34,35,36]. The effect of HZ on the risk of dementia was based on a meta-analysis of 57 studies [7], while the effect of RZV on dementia incidence was derived from previous vaccine-related epidemiological studies [17]. Furthermore, in the absence of RZV-specific longitudinal data for dementia-related mortality, this parameter was extrapolated from evidence based on the live-attenuated zoster vaccine (LZV) [4]. This proxy is considered conservative, as RZV has demonstrated superior clinical efficacy and more potent immunogenicity compared to LZV in preventing HZ, thereby likely offering greater reduction in the neuroinflammation linked to cognitive decline [20,37] (Table 1).

### 2.4. Vaccine Efficacy and Coverage

Vaccine efficacy of RZV against HZ was obtained from the vaccine package insert [13,14]. As the insert did not report efficacy against PHN or waning of vaccine protection, these parameters were derived from published economic evaluation models [21,23]. To estimate the potential public health impact under optimal implementation conditions, a vaccination coverage rate of 90% and a full two-dose completion rate were assumed. This scenario is consistent with the performance of other government-funded vaccines in China and serves as a benchmark for assessing the maximum health benefits of RZV [37,47] (Table 1).

### 2.5. Costs and Utilities

Costs included vaccination costs and disease-related costs. Vaccination costs comprised the vaccine acquisition cost, obtained from the China Pharmaceutical Pipeline Monitor, and a vaccination service fee per dose based on previous economic evaluations conducted in China [20,37]. This service fee encompasses the standardized clinical procedures associated with immunization, including pre-vaccination screening, vaccine administration, medical consumables, post-vaccination observation, and immunization information management services [48]. Disease-related costs for MCI, dementia, and HZ included direct medical costs, direct non-medical costs, and indirect costs. In the absence of localized data in China, MCI-related costs were derived from a nationwide South Korean study owing to the comparable disease patterns and socio-cultural structures within the East Asian context [40]. Dementia-related costs were obtained from a multicenter study of patients with Alzheimer’s disease in China [41]. Direct medical costs of HZ were based on electronic health record data [33], while indirect costs were informed by a Chinese community-based study [42]. All costs were converted to 2026 US dollars (1 USD = 7 RMB) and adjusted for inflation where necessary. Health utility parameters included age-specific utility values and health state-specific utility weights. Age-specific utilities were derived from the China National Health Service Survey [43]. The utility for MCI was sourced from a systematic review reporting health-related quality of life (HRQoL) in individuals with MCI [44]. The utility for dementia was derived from baseline 5-level EuroQoL 5 dimensions (EQ-5D-5L) measurements from an intervention study to represent the health status of individuals in a real-world setting [45]. Utility values for HZ and its complications were derived from a cross-sectional study of patients in China [46]. Health outcomes were expressed as quality-adjusted life years (QALYs). Both costs and utilities were discounted at an annual rate of 3% in accordance with World Health Organization recommendations [38,39] (Table 1).

### 2.6. Cost-Effectiveness Analyses

The incremental cost-effectiveness ratio (ICER) served as the primary outcome measure to compare the two-dose RZV vaccination strategy with no vaccination in immunocompetent adults aged 50 years with MCI. The ICER was calculated as the difference in costs divided by the difference in QALYs between the two strategies [49]. Secondary outcomes included the proportions of disease cases averted and the number needed to vaccinate (NNV) to prevent one case of HZ, PHN, and progression to dementia [50]. In the absence of an official cost-effectiveness threshold for vaccines in China, the 2024 Chinese gross domestic product (GDP) per capita (13,121 USD) [28] was utilized as the willingness-to-pay (WTP) threshold based on World Health Organization recommendations [38,39]. This approach has been widely adopted in economic evaluations conducted in similar settings [20,21,22,23,37,51]. An ICER below the GDP per capita was identified as cost-effective, whereas values between one and three times the GDP per capita were classified as acceptable. An ICER exceeding three times the GDP per capita was defined as not cost-effective [49].

### 2.7. Sensitivity Analyses

Sensitivity analyses were conducted to assess the robustness of the base-case results and to evaluate parameter uncertainty. Deterministic sensitivity analysis (DSA) was performed using one-way sensitivity analyses, in which individual parameters were varied across predefined ranges to assess their impact on the results. The findings were presented using tornado diagrams. Probabilistic sensitivity analyses (PSA) were conducted using 10,000 Monte Carlo simulations, in which all model parameters were varied simultaneously according to their respective probability distributions. The results were presented as a cost-effectiveness acceptability curve, illustrating the probability that the RZV strategy is cost-effective at different WTP thresholds. A scenario analysis was performed to address structural uncertainty regarding cognitive dynamics by allowing bidirectional transitions between normal cognition and MCI.

## 3. Results

### 3.1. Base-Case Analysis Results

Based on a simulated cohort of 1 million Chinese adults aged 50 years with MCI, the RZV strategy reduced disease burden across all evaluated outcomes compared to no vaccination. Over the lifetime horizon, RZV vaccination resulted in averted disease proportions of 54.64% for HZ, 97.58% for PHN, and 12.28% for dementia. This corresponded to a gain of 2.23 million QALYs. The incremental cost associated with RZV vaccination was 9386.36 million USD, resulting in an ICER of 4216.99 USD/QALY. Compared to the 2024 Chinese GDP per capita threshold (13,121 USD), the RZV strategy was considered cost-effective in adults aged 50 years with MCI. The NNV to prevent one case of HZ and PHN were 6.25 and 24.03, respectively. In addition, one case of progression to dementia was prevented for every 280.82 individuals vaccinated (Table 2).

### 3.2. One-Way Sensitivity Analyses

**Figure 2:**
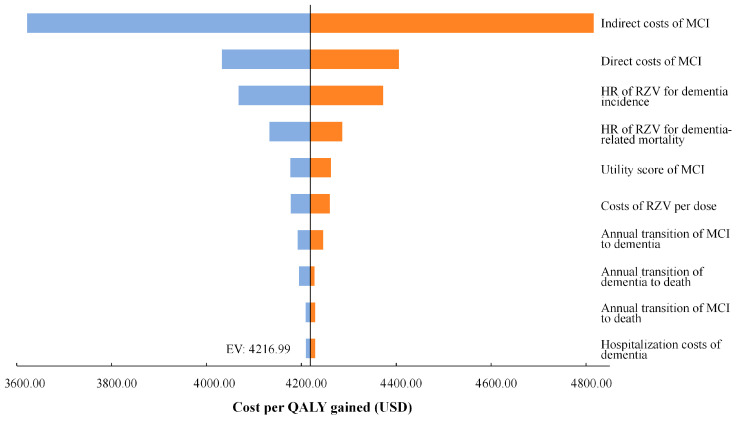
Tornado diagram of one-way sensitivity analysis of RZV versus no vaccination. MCI, mild cognitive impairment; HR, Hazard Ratio; QALY, quality-adjusted life-year; RZV, recombinant zoster vaccine.

One-way sensitivity analyses were conducted for all model inputs. The tornado diagram in Figure 2 presents the ten most influential parameters affecting the ICER. The results were most sensitive to the indirect cost of MCI, followed by the direct cost of MCI, the hazard ratio (HR) of RZV for dementia incidence, the HR for dementia-related mortality, the utility value of MCI, and the cost per dose of RZV. Transition probabilities from MCI to dementia, dementia to death, and MCI to death, as well as the indirect cost of dementia, also had moderate impacts on the ICER. Across all plausible parameter ranges, the ICER remained below the designated WTP threshold (13,121 USD/QALY). These findings indicate that the cost-effectiveness of RZV vaccination among adults aged 50 years with MCI is robust to variations in key epidemiological, cost, and utility parameters.

### 3.3. Probabilistic Sensitivity Analyses

**Figure 3:**
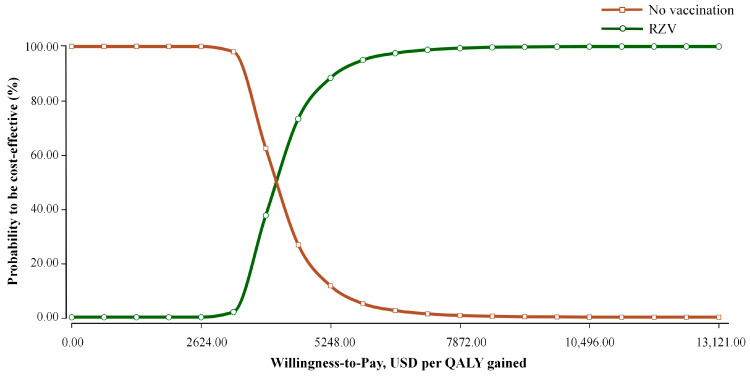
Cost-effectiveness acceptability curve of RZV versus no vaccination. QALY, quality-adjusted life-year; RZV, recombinant zoster vaccine.

The cost-effectiveness acceptability curve in Figure 3 illustrates the probability of each strategy being economically preferred across a range of WTP thresholds. The probability of RZV vaccination being cost-effective exhibited a positive correlation with the WTP threshold. At WTP values below 3936.30 USD/QALY, the no-vaccination strategy remained the preferred option in 62.40% of the simulations. The RZV strategy surpassed the no-vaccination strategy within the WTP interval of 3936.30 to 4592.35 USD/QALY. As the threshold increased to 5248.40 USD/QALY, the probability of RZV being cost-effective was 88.41%, which subsequently rose to 97.51% at a WTP of 6560.50 USD/QALY. At the specified cost-effectiveness threshold of 13,121 USD/QALY, the probability of RZV vaccination being cost-effective reached 100%. These results demonstrated the high robustness of the base-case findings despite joint parameter uncertainty.

### 3.4. Scenario Analysis

Scenario analysis results in Table 3 indicated that the RZV strategy remained cost-effective when cognitive reversion was included in the model. The ICER decreased from 4216.99 USD/QALY in the base case to 3182.28 USD/QALY, which was below the 2024 Chinese GDP per capita threshold of 13,121 USD. Vaccination resulted in an averted disease proportion of 15.71% for dementia progression, representing an increase from the 12.28% observed in the base case analysis. The NNV to prevent one case of HZ and PHN decreased to 5.49 and 22.62, respectively. One case of progression to dementia was prevented for every 275.63 individuals vaccinated, which was a reduction from the 280.82 individuals reported in the base case results. These findings indicated that the RZV strategy remained an efficient intervention when cognitive dynamics were integrated into the analysis.

## 4. Discussion

This study evaluated the health economic value of the RZV in adults aged 50 years with MCI from a societal perspective. In the base-case analysis, the ICER was 4216.99 USD/QALY, which was substantially below the threshold of the GDP per capita (13,121 USD/QALY), indicating that the RZV strategy is highly cost-effective. The public health benefits of RZV extend beyond the prevention of HZ and PHN, notably including a delay in progression from MCI to dementia. The NNV was 6.25 for HZ, 24.03 for PHN, and 280.82 for dementia progression. These findings indicated that vaccination in this high-risk population may yield substantial clinical and public health benefits.

Previous economic evaluations of RZV in general older populations have primarily focused on reductions in HZ and PHN incidence, often neglecting potential cognitive benefits [19,20,21,37]. Emerging evidence from natural experiments and large cohort studies has demonstrated that HZ vaccination is associated with reduced dementia incidence and mortality. A study conducted in Wales reported a 3.5% reduction in the risk of new dementia diagnoses over seven years [24]. Similarly, an Australian study observed an absolute reduction of 1.8% over a 7.4-year follow-up period and found that this protective effect was specific to dementia outcomes [11]. In addition, data from the Secure Anonymized Information Linkage (SAIL) databank indicated that vaccination reduced dementia-related mortality by 29.5% and all-cause mortality by 22.7% over nine years [4]. Incorporating these effects into the model allowed the capture of additional health gains beyond conventional dermatological outcomes, thereby increasing QALYs gained per unit cost. From a policy perspective, targeting the MCI population reflects a precision immunization strategy that enhances resource allocation efficiency. Compared with the general population, individuals with MCI have a substantially higher risk of progression to dementia [9,10,18], making them more responsive to preventive interventions. While the NNV for preventing HZ is relatively low in the general population, its impact on long-term care costs and productivity loss is limited. In contrast, among individuals with MCI, the high baseline risk of dementia progression and the associated indirect costs enable the benefits of vaccination to translate into substantial societal savings. The low ICER observed in this study reflects the economic advantage of targeting high-risk populations.

Several biological mechanisms may explain the observed association between HZ vaccination and dementia outcomes. Reactivation of VZV has been linked to long-term cognitive impairment through vasculopathy, characterized by ischemia, infarction, and hemorrhage involving both large and small vessels, resembling cerebrovascular pathology observed in Alzheimer’s disease [1,8,52,53,54]. VZV reactivation triggered neuroinflammatory cascades, promoted amyloid deposition, and induced secondary reactivation of latent herpes simplex virus (HSV) [52,55]. Genetic evidence localized to the 19q13 (APOE) region suggested that vascular pathologies mediated the relationship between polygenic risk and dementia. Such genetic susceptibility likely exacerbated the cognitive damage resulting from VZV-induced vasculopathy [56]. Beyond pathogen-specific protection, RZV may exert broader immunomodulatory effects that counteract immunosenescence and support neuroimmune function [24,57]. Vaccination during the MCI stage, a critical intervention window, may therefore help maintain neuroimmune homeostasis and delay disease progression.

One-way sensitivity analysis identified indirect costs of MCI as the most influential parameter, followed by direct medical costs and the HR for dementia incidence. This differs from models in general populations, where ICERs are typically most sensitive to HZ incidence, vaccine efficacy, and waning rates [21,22,23,37]. These findings underscore the central role of productivity loss and functional decline associated with dementia in economic evaluations. When indirect costs are included, the value of preventing dementia progression is substantially amplified. Probabilistic sensitivity analysis further demonstrated that the RZV strategy remained cost-effective in 100% of simulations at the WTP, indicating strong robustness of the findings. The scenario analysis addressed structural uncertainty by incorporating bidirectional transitions between normal cognition and MCI. While the base case analysis adopted a parsimonious unidirectional approach, the inclusion of cognitive reversion resulted in a more favourable ICER of 3182.28 USD per QALY. The NNV to prevent one case of dementia improved from 280.82 to 275.6, which indicated that the economic value of RZV was robust under different cognitive progression assumptions. This analysis showed that accounting for the possibility of cognitive stability and recovery further increased the efficiency of the vaccination strategy. By considering the full spectrum of cognitive dynamics, the model provided a conservative estimate of the intervention value.

Currently, RZV is not included in China’s national immunization program, and the high out-of-pocket cost may limit uptake among individuals with MCI. Given the economic vulnerability of this population, policy measures such as price negotiation, insurance reimbursement, and targeted public health programs should be considered to improve vaccine accessibility. Expanding access to RZV in high-risk populations could reduce both healthcare expenditures and long-term care burdens.

This study has several limitations. First, RZV was not compared with the domestic LZV (Changchun, China, Ganwei, Changchun Bcht) approved in China in 2023 due to the lack of robust long-term efficacy data, particularly for HZ, PHN, and cognitive outcomes. Second, some parameters were derived from other East Asian populations such as Japan and South Korea, which, despite disease patterns and socio-cultural similarities, may limit generalizability. In addition, the effect of RZV on dementia-related mortality was extrapolated from LZV data, which may underestimate its true survival benefits given its higher efficacy [20]. Third, the model assumed an optimal vaccination scenario with high coverage and full series completion to reflect the potential impact of a government-funded program. While this exceeds current uptake, it is consistent with other publicly funded vaccines in China and serves as a benchmark for maximum achievable benefit. Finally, although the vaccination service fee was included, broader programmatic costs such as cold-chain transportation and storage were not considered, potentially leading to a slight underestimation of total costs, and the model was not stratified by sex, such that sex-specific differences in epidemiology and outcomes were not explicitly captured.

Future research should focus on long-term follow-up of Chinese MCI cohorts to validate the causal relationship between VZV vaccination and cognitive outcomes. Economic evaluations that incorporate different public health interventions across population groups will assist resource allocation decisions beyond vaccine comparisons [58]. Future studies including age and sex heterogeneous populations are needed to assess cost-effectiveness across older age groups with differing baseline risks. As more data on domestic LZV becomes available, cost-effectiveness analyses across vaccine platforms will be needed to inform immunization strategies.

## 5. Conclusions

This study demonstrated that RZV vaccination in adults aged 50 years with MCI in China provides substantial epidemiological and economic benefits. The strategy reduces the incidence of HZ and PHN while delaying progression to dementia and improving quality of life. These findings provide important evidence to support immunization policy and targeted public health strategies aimed at reducing the dual burden of HZ and dementia. Policymakers should consider measures to improve vaccine accessibility among high-risk groups.

## Figures and Tables

**Table 1:** Parameters used in base-case and sensitivity analyses.

Parameters	Base Value	Range	Distribution	Source
Start age, years	50	-	-	-
Time horizon	Lifetime	-	-	-
Cycle length, years	1	-	-	-
Discount rate (cost)	0.03	0.00–0.05	Beta	[38,39]
Discount rate (QALY)	0.03	0.00–0.05	Beta	[38,39]
**Epidemiology**				
Annual all-cause mortality rate, %				[27,28]
50–59 years	0.37	±20%	Beta	
60–69 years	0.95	±20%	Beta	
≥70 years	4.87	±20%	Beta	
Annual transition of NC to MCI, %	19.09	17.75–20.43	Beta	[29]
Annual transition of MCI to NC, %	12.51	11.97–13.05	Beta	[29]
Annual transition of MCI to dementia, %	11.30	9.50–13.90	Beta	[5]
Annual transition of MCI to death, %	3.08	±20%	Beta	[30]
Annual transition of dementia to death, %	10.00	±20%	Beta	[31]
Annual incidence of HZ, %				[32]
50–59 years	0.69	0.29–1.08	Beta	
60–69 years	1.11	0.54–1.67	Beta	
≥70 years	1.15	0.50–2.06	Beta	
Annual recurrent rate of HZ, %				[33]
50–59 years	1.18	±20%	Beta	
60–69 years	1.63	±20%	Beta	
≥70 years	2.98	±20%	Beta	
Probability of PHN, %				[34,35,36]
50–59 years	6.30	5.20–7.40	Beta	
60–69 years	10.30	9.00–11.60	Beta	
≥70 years	20.30	9.50–37.80	Beta	
Annual recovery rate of PHN, %	70.00	±20%	Beta	[34,35,36]
Probability of other complications, %				[34,35,36]
50–59 years	3.20	2.40–4.00	Beta	
60–69 years	4.00	3.10–4.80	Beta	
≥70 years	3.40	2.80–5.10	Beta	
Annual case fatality rate of HZ, %				[21]
50–59 years	0.0007	±20%	Beta	
60–69 years	0.0013	±20%	Beta	
70–79 years	0.0056	±20%	Beta	
80–89 years	0.0366	±20%	Beta	
90–99 years	0.2002	±20%	Beta	
≥100 years	1.0352	±20%	Beta	
HR for dementia incidence after HZ	2.97	1.89–4.66	Log-normal	[7]
HR of dementia incidence after RZV	0.73	0.49–0.85	Log-normal	[4,17]
HR of dementia-related mortality after RZV	0.67	0.45–0.91	Log-normal	[4,17]
**Efficacy**				
RZV against HZ (2-dose, %)				Package insert
50–59 years	96.60	89.60–99.40	Beta	
60–69 years	97.40	90.10–99.70	Beta	
≥70 years	91.30	86.80–94.50	Beta	
RZV against PHN (2-dose, %)				[23]
50–69 years	98.90	94.00–100.00	Beta	
≥70 years	95.40	89.70–100.00	Beta	
Annual waning of RZV efficacy (2-dose, %)				[21]
50–69 years	1.50	0.00–3.40	Beta	
≥70 years	2.30	0.30–4.40	Beta	
**Cost (USD)**				
RZV (1-dose)	228.57	±20%	Gamma	Local data
Vaccination service (1-dose)	3.57	±20%	Gamma	[21,37]
Direct costs				
MCI	785.90	±20%	Gamma	[40]
Dementia				[41]
Outpatient costs	2501.18	±20%	Gamma	
Hospitalization costs	9692.09	±20%	Gamma	
Other medical costs	468.97	±20%	Gamma	
Other nonmedical costs	5087.01	±20%	Gamma	
Uncomplicated HZ				[33]
Outpatient costs	81.28	2.10–882.70	Gamma	
Hospitalization costs	768.88	81.14–1808.43	Gamma	
Complicated HZ				[33]
Outpatient costs	67.43	2.67–381.65	Gamma	
Hospitalization costs	1550.40	277.38–3863.67	Gamma	
Indirect cost				
MCI	2539.00	±20%	Gamma	[40]
Dementia	10,031.13	±20%	Gamma	[41]
Uncomplicated HZ	111.06	±20%	Gamma	[21,42]
Complicated HZ	265.28	±20%	Gamma	[21,42]
**Utility**				
50–59 years	0.950	0.949–0.951	Beta	[43]
60–69 years	0.899	0.897–0.901	Beta	[43]
≥70 years	0.815	0.777–0.861	Beta	[43]
MCI	0.850	0.780–0.900	Beta	[44]
Dementia	0.400	0.352–0.448	Beta	[45]
Uncomplicated HZ	0.850	0.700–0.940	Beta	[46]
PHN	0.740	0.650–0.830	Beta	[46]
Other complications	0.700	0.490–0.790	Beta	[46]

For parameters lacking published confidence intervals or robust empirical ranges, deterministic sensitivity analysis was performed using a ±20% variation around base-case values. Probabilities and utilities were modelled using beta distributions, cost parameters using gamma distributions, and hazard ratios using log-normal distributions. HZ, herpes zoster; PHN, postherpetic neuralgia; NC, normal cognition; MCI, mild cognitive impairment; HR, Hazard Ratio; QALY, quality-adjusted life-year; RZV, recombinant zoster vaccine.

**Table 2:** Base-case analysis results of RZV versus no vaccination.

	No Vaccination	RZV Vaccination
**Disease events**		
HZ cases	292,878	132,848
PHN cases	42,652	1030
Dementia cases	29,006	25,445
**Averted disease (%)**		
HZ	-	54.64
PHN	-	97.58
Dementia	-	12.28
Costs (million USD)	55,358.95	64,089.21
QALYs (million)	12.36	15.36
Incremental Costs (million USD)	-	9386.36
Incremental QALYs (million)	-	2.23
ICER (USD/QALY)	-	4216.99
**NNV**		
HZ	-	6.25
PHN	-	24.03
Dementia	-	280.82

HZ, herpes zoster; PHN, postherpetic neuralgia; ICER, incremental cost-effectiveness ratio; QALY, quality-adjusted life-year; RZV, recombinant zoster vaccine; NNV, number needed to vaccinate to prevent one case.

**Table 3:** Scenario analysis results of RZV versus no vaccination incorporating reversion from MCI to normal cognition.

	No Vaccination	RZV Vaccination
**Disease events**		
HZ cases	301,953	119,918
PHN cases	44,691	484
Dementia cases	23,096	19,468
**Averted disease (%)**		
HZ	-	60.29
PHN	-	98.92
Dementia	-	15.71
Costs (million USD)	42,908.45	50,882.16
QALYs (million)	15.15	17.66
Incremental Costs (million USD)	-	7973.71
Incremental QALYs (million)	-	2.51
ICER (USD/QALY)	-	3182.28
**NNV**		
HZ	-	5.49
PHN	-	22.62
Dementia	-	275.63

Scenario analysis addressed structural uncertainty by allowing bidirectional transitions between MCI and normal cognition. Reversion from dementia to any other cognitive state was not modelled to maintain clinical validity. Within the normal cognition stage, four health states were specified, including NC without HZ (NC_Well), acute HZ (NC_HZ), PHN (NC_PHN), and recovery following HZ (NC_Post_HZ). HZ, herpes zoster; PHN, postherpetic neuralgia; MCI, mild cognitive impairment; HR, Hazard Ratio; ICER, incremental cost-effectiveness ratio; QALY, quality-adjusted life-year; RZV, recombinant zoster vaccine; NNV, number needed to vaccinate to prevent one case.

## Data Availability

Data were retrieved from the articles mentioned in the references.
